# Pelargonidin-3-*O*-glucoside and its metabolites have modest anti-inflammatory effects in human whole blood cultures

**DOI:** 10.1016/j.nutres.2017.09.006

**Published:** 2017-10

**Authors:** Anna M. Amini, Karolin Muzs, Jeremy PE. Spencer, Parveen Yaqoob

**Affiliations:** Department of Food and Nutritional Sciences, University of Reading, Whiteknights, PO Box 226, Reading RG6 6AP, UK

**Keywords:** Anthocyanin, Inflammation, Phagocytosis, Primary cell culture, Strawberry, 4-HBA, 4-hydroxybenzoic acid, FCS, fetal calf serum, IL, interleukin, LPS, lipopolysaccharide, PCA, protocatechuic acid, Pg-3-glc, pelargonidin-3-*O*-glucoside, PGA, phloroglucinaldehyde, TNF-*α*, tumor necrosis factor-*α*

## Abstract

This study hypothesized that the predominant strawberry anthocyanin, pelargonidin-3-*O*-glucoside (Pg-3-glc), and 3 of its plasma metabolites (4-hydroxybenzoic acid, protocatechuic acid, and phloroglucinaldehyde [PGA]) would affect phagocytosis, oxidative burst, and the production of selected pro- and anti-inflammatory cytokines in a whole blood culture model. For the assessment of phagocytosis and oxidative burst activity of monocytes and neutrophils, whole blood was preincubated in the presence or absence of the test compounds at concentrations up to 5 *μ*mol/L, followed by analysis of phagocytic and oxidative burst activity using commercially available test kits. For the cytokine analysis, diluted whole blood was stimulated with lipopolysaccharide in the presence or absence of the test compounds at concentrations up to 5 *μ*mol/L. Concentrations of selected cytokines (tumor necrosis factor-*α*, interleukin [IL]-1*β*, IL-6, IL-8, and IL-10) were determined using a cytometric bead array kit. There were no effects of any of the test compounds on phagocytosis of opsonized or nonopsonized *Escherichia coli* or on oxidative burst activity. Pg-3-glc and PGA at 0.08 *μ*mol/L increased the concentration of IL-10 (*P* < .01 and *P* < .001, respectively), but there was no effect on tumor necrosis factor-*α*, IL-1*β*, IL-6, and IL-8, and there were no effects of the other compounds. In conclusion, this study demonstrated a lack of effect of these compounds on the opsonization, engulfment, and subsequent destruction of bacteria. Pg-3-glc and PGA, at physiologically relevant concentrations, had anti-inflammatory properties; however, effects were modest, only observed at the lowest dose tested and limited to IL-10.

## 1 Introduction

Anthocyanins are polyphenols which are abundant in berry fruits and which may convey health benefits to humans, including cardiovascular disease prevention, obesity control, alleviation of diabetes, improvement of vision and memory, and increased immune defenses [1], [2]. An anthocyanin-rich elderberry extract was demonstrated to exert antimicrobial and antiviral activity in vitro toward human pathogenic respiratory bacteria and influenza viruses, although mechanisms were unclear [3]. Leukocytes play a crucial role in pathogen defense [4], and several leukocyte functions have been reported to be influenced by flavonoids, although some of the data are conflicting. Blueberries [5], an anthocyanin-rich juice [6], and purple sweet potato leaves [7] were reported to increase numbers and activity of natural killer cells [5], but there was no effect of red wine, dealcoholized red wine, or red grape juice [8], [9]. Similarly, lymphocyte proliferation and interleukin (IL)-2 secretion by activated lymphocyte were increased upon consumption of an anthocyanin-rich juice [6] and purple sweet potato leaves [7] but not affected by red wine, dealcoholized red wine, or red grape juice [8], [9]. Red wine, dealcoholized red wine, or red grape juice also had no effect on phagocytosis by neutrophils and monocytes [8], [9]. In an animal model, red wine anthocyanins increased phagocytic activity at 25 and 50 mg/kg body weight but decreased phagocytic activity at higher doses [10]. In mice, pyogallol-type green tea polyphenols increased phagocytic activity in vitro [11], and a polyphenol-rich cereal fraction increased phagocytic activity and increased production of reactive oxygen species and superoxide anion [12], but a number of anthocyanin- and flavonoid-rich fruits were reported to diminish reactive oxygen species production [13]. There are a lack of evidence from human studies and limited and conflicting data regarding the influence of flavonoids on the phagocytic process.

Several cell culture studies have explored the effect of anthocyanins to modulate cytokine production and other parameters of immune function, but most were conducted using the unmetabolized parent anthocyanins, often at high doses [3], [10], [13], [14], [15], [16], [17], [18], [19], [20], [21], [22], [23], [24], [25], [26], which may not be physiologically relevant. Strawberries constitute a popular fruit, and they are particularly rich in anthocyanins, predominantly pelargonidin-3-*O*-glucoside (Pg-3-glc) [27]. Glucuronidated pelargonidin has been reported as the predominant metabolite in 3 pharmacokinetic studies [28], [29], [30], but there is ambiguity regarding the position of glucuronidation, and glucuronidated pelargonidin compounds are currently commercially unavailable and hence cannot be tested in cell culture models. 4-Hydroxybenzoic acid (4-HBA) and protocatechuic acid (PCA) have also been reported in plasma following strawberry consumption in low–micromole per liter concentration (0.1–2 *μ*mol/L) [30], [31], [32]. In addition, it is likely that phloroglucinaldehyde (PGA) might appear in plasma following strawberry consumption, as it is an A-ring degradant, reported in plasma upon anthocyanin consumption in low– to high–nanomole per liter concentration (20-600 nmol/L) [33], [34]. It is important to note in this context that 4-HBA, PCA, and PGA are not pelargonidin-specific metabolites. Their presence in plasma has been reported following ingestion of other flavonoids, and they are also naturally present in several other dietary sources. However, there are very little data on the effects of these physiologically relevant compounds. Furthermore, most in vitro work has been conducted using cell lines, but whole blood cultures more closely represent physiological conditions [35], [36]. This study therefore characterized the effect of the parent anthocyanin Pg-3-glc and 3 physiologically relevant plasma metabolites on phagocytosis, oxidative burst, and the production of selected pro- and anti-inflammatory cytokines (tumor necrosis factor-*α* [TNF-*α*], IL-1*β*, IL-6, IL-8, and IL-10) in a whole blood culture model to test our hypothesis that modulations would be observed.

## 2 Methods and materials

### 2.1 Subjects

Ten healthy volunteers (8 women and 2 men) were recruited for this pilot study. Inclusion criteria included the following: 40-65 years old; good general health; and absence of diabetes, cancer, liver cirrhosis, asplenia, other acquired or congenial immunodeficiency, HIV, or any kind of inflammatory, autoimmune, or connective tissue disease. The exclusion criteria were use of anti-inflammatory or immunomodulating medication, use of antibiotics within 3 months, vaccination within 3 months, participation in another drug or nutritional research study within 3 months, and alcoholism or drug misuse. Subjects were asked to follow a low-flavonoid diet for 24 hours prior to the blood sample collection. Volunteers arrived following a 12-hour fast to the Hugh Sinclair Unit of Human Nutrition of the University of Reading, and blood was collected into sodium heparin vacutainer tubes (Greiner Bio-One Ltd, Gloucestershire, UK). Written informed consent was obtained from all subjects. The work was conducted according to the guidelines laid down in the Declaration of Helsinki and approved by the University of Reading Research Ethics Committee (Project reference 10/05).

### 2.2 Materials

Pg-3-glc was purchased from Extrasynthese (Genay, France). PCA (3,4-dihydroxybenzoic acid), 4-HBA, PGA (2,4,6-trihydroxybenzaldehyde), lipopolysaccharides from *Escherichia coli* (LPS), methanol, and formic acid were purchased from Sigma-Aldrich (Dorset, United Kingdom). RPMI 1640 culture medium, fetal calf serum (FCS), and antibiotics (penicillin and streptomycin) were purchased from Lonza (Basel, Switzerland). The cytometric bead array kit to analyze cytokine concentrations was purchased from BD Biosciences (Oxford, United Kingdom). Phagoburst and Phagotest kits as well as the opsonized *E coli* bacteria were manufactured by Glycotope Biotechnology (Heidelberg, Germany). Pg-3-glc, PCA, 4-HBA, and PGA were dissolved in acidified methanol (2% formic acid) to a concentration of 10 mmol/L and stored at −70°C and away from light. Further dilutions of the test compounds were prepared freshly on each study day by dilution in RPMI medium with added FCS and antibiotics. RPMI culture medium was stored at 4°C. FCS and antibiotics were defrosted upon delivery, aliquoted, and stored at −20°C until use.

### 2.3 Whole blood culture for phagocytosis and oxidative burst capacity

Heparinized whole blood samples were preincubated with the test compounds (Pg-3-glc, PCA, 4-HBA, PGA, or RPMI 1640 medium as control) at 4 different concentrations (0.08, 0.31, 1.25, and 5 *μ*mol/L) at 37°C for 4 hours in 15 × 75–mm tubes.

### 2.4 Whole blood culture for cytokine analysis

Heparinized whole blood was diluted 6:10 with RPMI 1640 medium supplemented with FCS and antibiotics. The diluted blood (1 mL/well) was placed into 24-well tissue culture plates. Working solutions of the test compounds were added to provide final concentrations of 0.08, 0.31, 1.25, and 5 *μ*mol/L. Respective volumes of RPMI 1640 medium were added to control cultures (no polyphenols). LPS (1-*μ*g/mL final concentration) was added to stimulate cytokine production. Cultures were incubated at 37°C in a 5% CO_2_ atmosphere for 24 hours. At the end of the culture period, plates were centrifuged at 260*g* for 5 minutes. Culture supernatants were collected and stored in aliquots at −20°C until analysis.

### 2.5 Measurement of leukocyte phagocytosis and oxidative burst capacity

Phagocytic and oxidative burst activities of monocytes and neutrophils were analyzed using commercially available test kits (Phagotest and Phagoburst) following the instructions of the manufacturer. The percentage of neutrophils or monocytes engaged in phagocytosis of *E coli* bacteria (opsonized and nonopsonized) and oxidative burst activity and the mean fluorescence intensity were acquired on a BD FACS Canto II flow cytometer. Data were analyzed using DIVA software.

### 2.6 Measurement of cytokine concentrations

Concentrations of TNF-*α*, IL-1*β*, IL-6, IL-8, and IL-10 in the culture supernatants were measured using a cytometric bead array kit from BD Biosciences (Oxford, United Kingdom) according to the manufacturer's instructions. The intensity of the fluorescence signal was acquired on a BD FACS Canto II flow cytometer, and data were analyzed using the BD FCAP Array v3 software. Limits of detection of the cytokine assays are 0.13 pg/mL (IL-10), 1.2 pg/mL (TNF-*α* and IL-8), 1.6 pg/mL (IL-6), and 2.3 pg/mL (IL-1*β*).

### 2.7 Statistical analyses

Results are expressed as percentage of phagocytic activity/oxidative burst activity/cytokine production vs control (no polyphenols) and shown as means with their standard deviations (SD). One-way analysis of variance (ANOVA) was performed, followed by Dunnett as post hoc analysis vs control group where appropriate. Statistical analysis was performed using SPSS 21 (IBM Corporation, Armonk, NY, USA), and a lowered *P* < .01 was considered significant to account for multiple comparisons.

## 3 Results

### 3.1 Effects of Pg-3-glc, PCA, 4-HBA, and PGA on phagocytic and oxidative burst activity

**Fig. 1:**
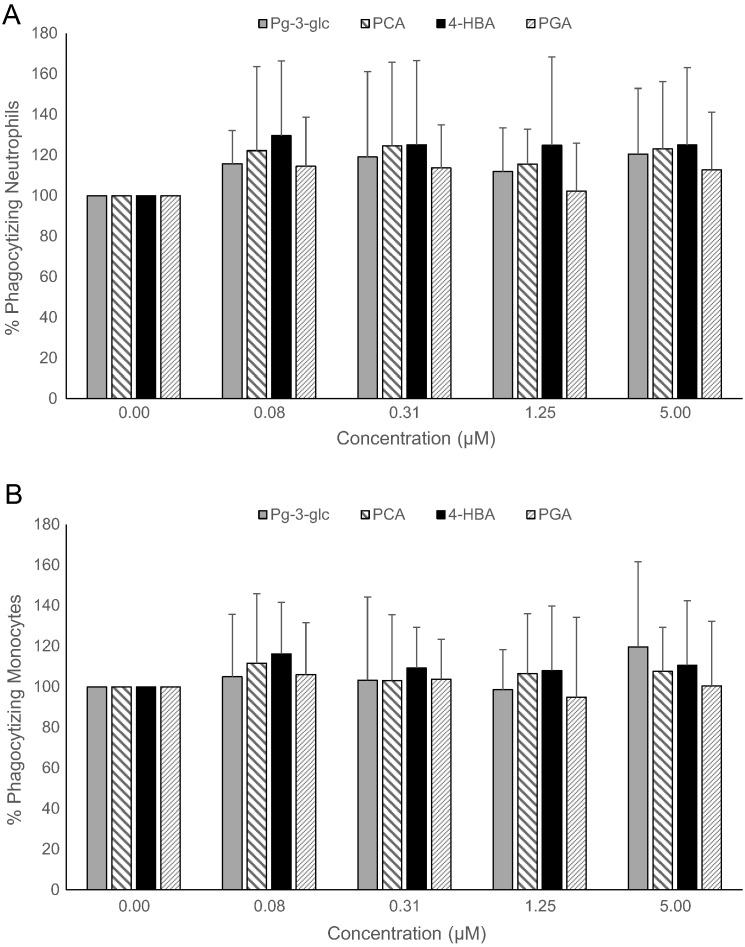
Effect of Pg-3-glc, PCA, 4-HBA, and PGA on phagocytic activity of nonopsonized *E coli* bacteria in human neutrophils (A) and monocytes (B). Human whole blood (n = 10) was treated with Pg-3-glc, PCA, 4-HBA, PGA, or vehicle control at concentrations of 0-5 *μ*mol/L, for 4 hours at 37°C. Phagocytic activity was analyzed using the Phagotest test kit. Results are expressed as percentage of phagocytic activity vs control (no polyphenols). Data are represented as the means ± SD. Data were analyzed by 1-way ANOVA, and a lowered *P* < .01 was considered significant to account for multiple comparisons. There were no statistically significant changes.

**Fig. 2:**
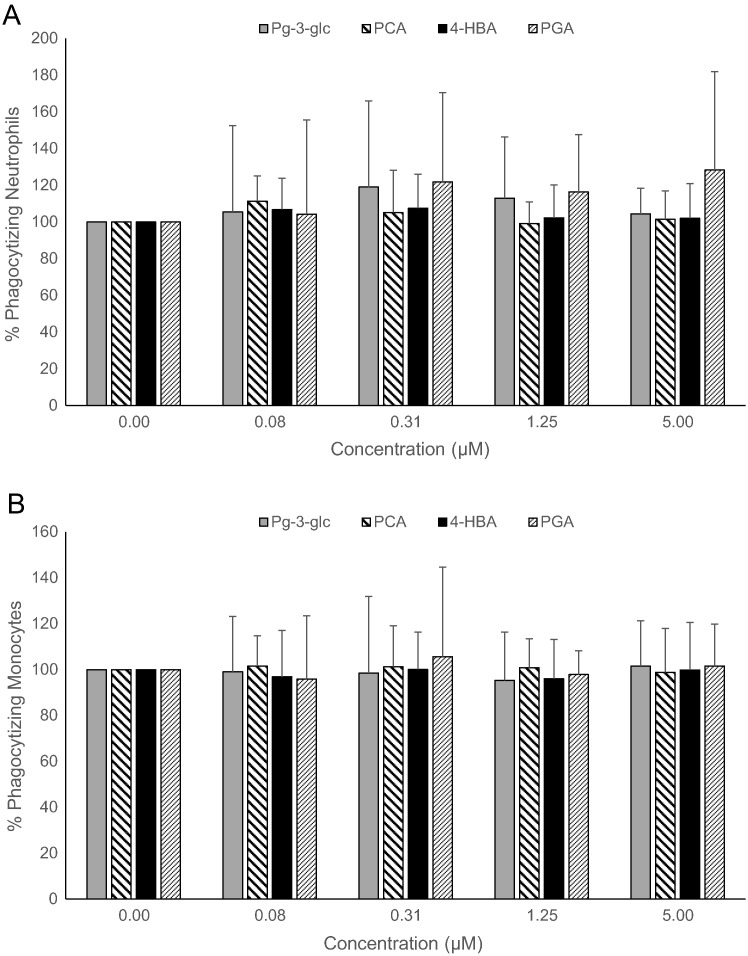
Effect of Pg-3-glc, PCA, 4-HBA, and PGA on phagocytic activity of opsonized *E coli* bacteria in human neutrophils (A) and monocytes (B). Human whole blood (n = 10) was treated with Pg-3-glc, PCA, 4-HBA, PGA, or vehicle control at concentrations of 0-5 *μ*mol/L, for 4 hours at 37°C. Phagocytic activity was analyzed using the Phagotest test kit. Results are expressed as percentage of phagocytic activity vs control (no polyphenols). Data are represented as the means ± SD. Data were analyzed by 1-way ANOVA, and a lowered *P* < .01 was considered significant to account for multiple comparisons. There were no statistically significant changes.

**Fig. 3:**
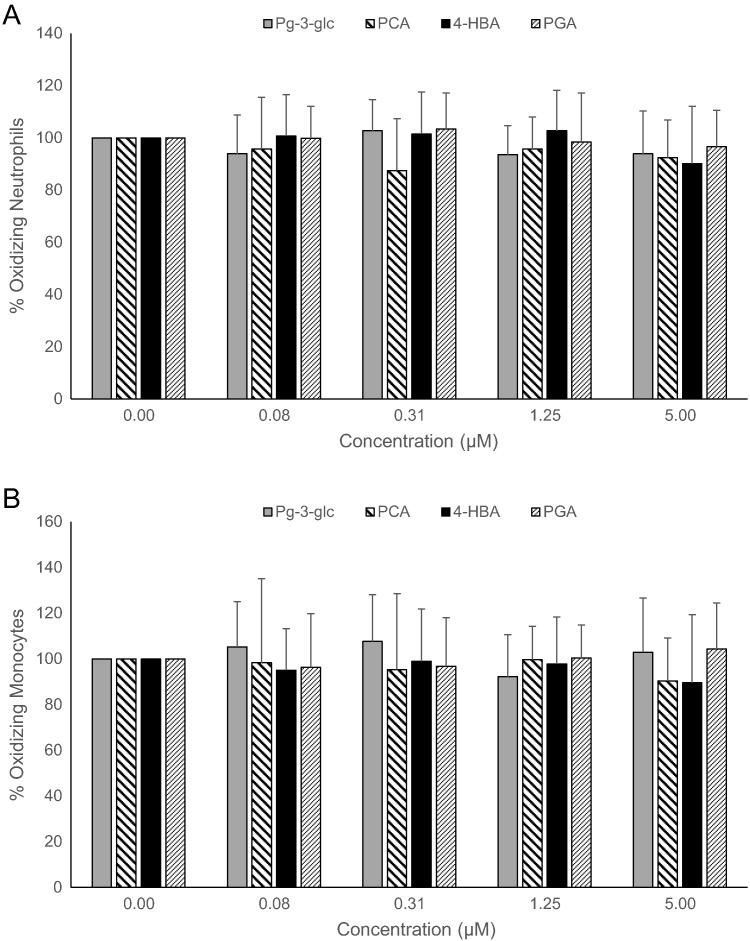
Effect of Pg-3-glc, PCA, 4-HBA, and PGA on oxidative burst activity in human neutrophils (A) and monocytes (B). Human whole blood (n = 10) was treated with Pg-3-glc, PCA, 4-HBA, PGA, or vehicle control at concentrations of 0-5 *μ*mol/L, for 4 hours at 37°C. Oxidative burst activity was analyzed using the Phagoburst test kit. Results are expressed as percentage of oxidative burst activity vs control (no polyphenols). Data are represented as the means ± SD. Data were analyzed by 1-way ANOVA, and a lowered *P* < .01 was considered significant to account for multiple comparisons. There were no statistically significant changes.

None of the test compounds significantly affected the overall percentage of neutrophils or monocytes engaged in phagocytosis of opsonized or nonopsonized *E coli* bacteria and their oxidative burst activity (Fig. 1, Fig. 2, Fig. 3). A high degree of intersubject variability was notable. Furthermore, there were no significant effects of any of the test compounds on the mean fluorescence intensity, which indicates degree of phagocytic/oxidative burst activity (data not shown).

### 3.2 Effects of Pg-3-glc, PCA, 4-HBA, and PGA on cytokine production

Stimulation with LPS increased IL-1*β* production 11-fold, TNF-*α* production 9-fold, IL-6 production 2-fold, IL-8 production 1-fold, and IL-10 production 5-fold. Pg-3-glc and PGA at the lowest dose tested (0.08 *μ*mol/L) significantly increased IL-10 production compared with the control cultures (*P* < .01 and *P* < .001, respectively; Table 1). There was no significant effect of any tested compound, at concentrations up to 5 *μ*mol/L, on the production of IL-1*β*, TNF-*α*, IL-6, or IL-8 by human whole blood cultures (Table 1).

**Table 1:** Effect of Pg-3-glc, PCA, 4-HBA, and PGA on IL-1*β*, TNF-*α*, IL-6, IL-8, and IL-10 production in human whole blood cultures

Cytokine production (% of control)	Pg-3-glc					PCA					4-HBA					PGA				
	0 *μ*mol/L	0.08 *μ*mol/L	0.31 *μ*mol/L	1.25 *μ*mol/L	5.00 *μ*mol/L	0 *μ*mol/L	0.08 *μ*mol/L	0.31 *μ*mol/L	1.25 *μ*mol/L	5.00 *μ*mol/L	0 *μ*mol/L	0.08 *μ*mol/L	0.31 *μ*mol/L	1.25 *μ*mol/L	5.00 *μ*mol/L	0 *μ*mol/L	0.08 *μ*mol/L	0.31 *μ*mol/L	1.25 *μ*mol/L	5.00 *μ*mol/L
IL-1*β*	100	95.8 ± 11.2	96.6 ± 11.1	93.0 ± 10.4	90.3 ± 13.8	100	99.0 ± 7.6	99.4 ± 7.1	99.8 ± 10.7	96.8 ± 9.8	100	91.2 ± 7.9	92.9 ± 7.6	90.8 ± 7.6	92.4 ± 17.0	100	93.4 ± 14.6	94.4 ± 8.7	98.4 ± 11.2	108.4 ± 13.7
TNF-*α*	100	100.2 ± 26.1	96.9 ± 14.6	96.1 ± 12.0	96.6 ± 12.0	100	108.0 ± 13.5	95.0 ± 16.1	95.3 ± 20.3	97.7 ± 16.7	100	101.0 ± 8.8	99.6 ± 8.8	98.4 ± 9.2	103.8 ± 22.2	100	95.6 ± 26.8	102.0 ± 19.1	100.2 ± 14.9	98.4 ± 13.3
IL-6	100	98.5 ± 5.4	93.9 ± 6.7	94.7 ± 9.1	93.5 ± 9.2	100	102.0 ± 10.6	94.8 ± 9.8	94.6 ± 10.5	95.3 ± 7.0	100	96.0 ± 7.0	94.4 ± 7.2	94.7 ± 9.2	98.7 ± 8.7	100	100.6 ± 11.6	96.0 ± 9.7	97.3 ± 9.4	93.2 ± 8.6
IL-8	100	106.2± 14.5	93.2 ± 10.4	89.8± 10.7	88.9 ± 11.0	100	106.0 ± 23.6	89.5 ± 15.0	89.8 ± 14.7	90.3 ± 13.5	100	108.5 ± 21.0	98.1 ± 15.6	95.9 ± 14.9	95.3 ± 12.5	100	109.3 ± 13.8	96.4 ± 10.2	94.9 ± 11.9	103.8 ± 15.4
IL-10	100^a^	118.0 ± 22.6^b^	98.5 ± 6.3	98.2 ± 7.6	100.1 ± 6.4	100	107.1 ± 14.4	97.4 ± 7.0	101.5 ± 5.9	102.7 ± 10.5	100	103.4 ± 11.1	97.0 ± 7.6	95.0 ± 8.1	97.2 ± 10.9	100^a^	123.8 ± 25.6	104.0 ± 7.8	97.1 ± 6.9^c^	98.6 ± 10.8

Human whole blood (n = 10) was treated with Pg-3-glc, PCA, 4-HBA, PGA, or vehicle control at concentrations of 0-5 *μ*mol/L, prior to LPS stimulation (1 *μ*g/mL), and incubated for 24 hours at 37°C. Cytokine concentrations in the culture supernatants were measured using a cytometric bead array kit. Results are expressed as percentage of cytokine concentration vs control (no polyphenols). Data are represented as the means ± SD. Data were analyzed by 1-way ANOVA and Dunnett post hoc analysis, where applicable, and a lowered *P* < .01 was considered significant to account for multiple comparisons. Statistically significant differences are denoted as ^a^*P* < .01 (1-way ANOVA), ^b^*P* < .01 vs control (Dunnett post hoc analysis), and ^c^*P* < .001 vs control (Dunnett post hoc analysis).

## 4 Discussion

The current study investigated, for the first time, the effects of the strawberry-derived anthocyanin Pg-3-glc and 3 of its physiologically relevant plasma metabolites on phagocytosis, oxidative burst, and the production of selected pro- and anti-inflammatory cytokines by whole blood cultures, and our hypothesis was only partially supported by the results. There were no effects of any of the test compounds on phagocytosis or on oxidative burst activity. In agreement with these data, some human intervention studies report no significant effects of anthocyanin-rich red wine, red grape juice [8], [9], or quercetin [37], [38], [39], [40] on phagocytic ability of monocytes and neutrophils. However, higher doses of red wine anthocyanins did increase phagocytic activity in mice [10], suggesting that the outcomes of animal and human studies may differ and that dose might be important.

Once pathogens are engulfed by phagocytes (phagocytosis), they are destroyed in part by the production of reactive oxygen metabolites in a process termed *oxidative burst* [4]. In the current study, contrary to our hypothesis, there were no effects of any of the test compounds on oxidative burst activity upon *E coli* stimulation. In contrast, a reduction in hydrogen peroxide production was reported by a raspberry fruit extract in phorbol-12-myristate-13-acetate–stimulated J774 murine macrophages, but the effects were only observed at higher extract concentrations and were less pronounced in arachidonic acid–stimulated macrophages [41]. Similarly, diminished production of reactive oxygen species production was reported by a number of anthocyanin- and flavonoid-rich fruits by opsonized zymosan-activated phagocytes, but no effects were observed with phorbol-12-myristate-13-acetate as stimulus [13]. This study used higher polyphenol doses compared with the current experiment, where doses may have been too low to have an effect. The 2 studies have also used a different technique for reactive oxygen species measurement. Another important consideration that may contribute to discrepant findings between studies might be the nature of the stimulus used. Emerging data suggest that the phagocytic immune response is governed by the type of stimulus [42]. It is important to note that interpretation of oxidative burst data is not straightforward. On the one hand, oxidative burst is involved in the destruction of pathogens upon phagocytosis and thus represents a critical component of immune defense [4], but it can also be harmful to tissues and contribute to the pathogenesis of chronic health conditions [13], especially if there are insufficient antioxidant defenses.

Cytokines are a critical component of immune defense, but, on the other hand, inappropriate or excessive production of TNF-*α*, IL-1*β*, IL-6, and IL-8 has been linked with the pathogenesis of a number of chronic inflammatory diseases [4]. IL-10, on the other hand, is a predominantly anti-inflammatory cytokine and would be expected to be associated with reduced atherosclerosis by suppressing macrophage activation and inhibiting several proinflammatory cytokines, chemokines, and growth factors [43]. In the current experiment, the increased IL-10 production by Pg-3-glc and PGA could be interpreted as a modest anti-inflammatory effect, and the original hypothesis is therefore partially upheld [43]. Interestingly, in the current experiment, the increase in IL-10 by Pg-3-glc and PGA was only observed at the lowest dose tested (0.08 *μ*mol/L). Importantly, these doses are physiologically relevant. PGA and Pg-3-glc were reported in plasma upon anthocyanin consumption at 5-600 nmol/L [29], [30], [33], [34]. Although the validity and interpretation of this effect remain to be confirmed, it could indicate the presence of an inverted U-shaped response curve. Previously, trends for (inverted) U-shaped associations were observed in a cell model testing vanillic acid and heme oxygenase-1 protein expression [44] in a human intervention study between blueberry beverage consumption and flow-mediated dilation [45] and in an epidemiological study between tea consumption and coronary heart disease mortality [46]. To our knowledge, no other studies have investigated the effect of PGA on IL-10 production by monocytes or macrophages. There were no effects of any of the other test compounds on IL-10 levels, which are in line with results on the effect of PCA in human monocyte–derived dendritic cells [25] and PCA and 4-HBA in THP-1 monocytes [47]. In the latter study, Pg-3-glc did not alter IL-10 production, which is in contrast to the current data. However, that study only tested a 1-*μ*mol/L dose, and the lack of effect was consistent with the observation that Pg-3-glc at 0.31-5.00 *μ*mol/L had no effect in the current study.

There is evidence from in vitro studies to suggest that particular structural characteristics might be required for phagocytosis-enhancing effects. A cell line study (using 1,25-dihydroxyvitamin D3–differentiated HL60 cells) concerned with the effect of green tea polyphenols suggested that a pyrogallol-type B-ring and/or a galloyl group is required to increase phagocytic activity [11]. These structural characteristics were absent from the tested compounds in the current studies. However, this observation was only based on a screen of 6 compounds and therefore requires confirmation, ideally in screening studies with a larger number of related compounds to clearly identify chemical structures or properties required for phagocytosis-enhancing effects.

A limitation of the present experiment is that although subjects were asked to follow a low-flavonoid diet for 24 hours prior to the blood sample collection, it cannot be excluded that some phenolic acids were present in circulation, which could have contributed to the variability and/or lack of effect.

In conclusion, there was no effect of the strawberry-derived anthocyanin Pg-3-glc or 3 of its physiologically relevant plasma metabolites on phagocytosis or oxidative burst activity in an in vitro human whole blood culture model. The data suggest that PGA and Pg-3-glc at physiologically attainable concentrations may possess anti-inflammatory properties through modulation of IL-10 production, which could contribute to protective effects in inflammatory diseases, although the magnitude of the effects appears to be modest and was only observed at the lowest dose tested (0.08 *μ*mol/L). None of the test compounds had any effect on IL-1*β*, TNF-*α*, IL-6, and IL-8. Subsequent studies should explore other immunomodulatory effects of dietary anthocyanins.
